# Analysis of a conserved cellulase transcriptional regulator reveals inducer-independent production of cellulolytic enzymes in *Neurospora crassa*

**DOI:** 10.1002/mbo3.94

**Published:** 2013-06-14

**Authors:** Samuel T Coradetti, Yi Xiong, N Louise Glass

**Affiliations:** Plant and Microbial Biology Department, The University of CaliforniaBerkeley, CA, 94720-3102

**Keywords:** *Aspergillus*, biofuels, cellulase, *Neurospora*, transcriptional regulation

## Abstract

Cellulose is recalcitrant to deconstruction to glucose for use in fermentation strategies for biofuels and chemicals derived from lignocellulose. In *Neurospora crassa*, the transcriptional regulator, CLR-2, is required for cellulolytic gene expression and cellulose deconstruction. To assess conservation and divergence of cellulase gene regulation between fungi from different ecological niches, we compared *clr-2* function with its ortholog (*clrB*) in the distantly related species, *Aspergillus nidulans*. Transcriptional profiles induced by exposure to crystalline cellulose were similar in both species. Approximately 50% of the cellulose-responsive genes showed strict dependence on functional *clr-2/clrB*, with a subset of 28 genes encoding plant biomass degrading enzymes that were conserved between *N. crassa* and *A. nidulans*. Importantly, misexpression of *clr-2* under noninducing conditions was sufficient to drive cellulase gene expression, secretion, and activity in *N. crassa*, to a level comparable to wild type exposed to Avicel. However, misexpression of *clrB* in *A. nidulans* was not sufficient to drive cellulase gene expression under noninducing conditions, although an increase in cellulase activity was observed under crystalline cellulose conditions. Manipulation of *clr-2* orthologs among filamentous fungi may enable regulated cellulosic enzyme production in a wide array of culture conditions and host strains, potentially reducing costs associated with enzyme production for plant cell wall deconstruction. However, this functionality may require additional engineering in some species.

## Introduction

Saprotrophic fungal species are major agents of plant decay in the environment (Kominkova et al. 2000; Hieber and Gessner 2002), with influences on the global carbon cycle (Heimann and Reichstein 2008), local nutrient flows (Frey et al. 2003), and the historic accumulation of fossil fuel deposits (Floudas et al. 2012). Depolymerization of cellulose for biotechnological applications is currently assisted by large-scale production of fungal enzyme mixtures and their application to pretreated plant biomass. However, commercially available enzyme mixtures for cellulose degradation remain prohibitively expensive for current saccharification processes (Margeot et al. 2009). A greater understanding of fundamental aspects of fungal enzyme synthesis and secretion is required, including defining regulatory networks that control enzyme production. Regulated control of cellulase gene expression across divergent fungal species would allow for high productivity of varied enzyme mixtures under a range of growth conditions and media compositions.

To elucidate the fungal potential for plant cell wall deconstruction and explore how environmental factors affect the production of enzymes mediating decay, we study regulatory mechanisms associated with cellulose degradation in two distantly related model filamentous fungi, *Neurospora crassa* and *Aspergillus nidulans*. In nature, *N. crassa* is associated with plant material that has been killed by fire (Perkins and Turner 1988). *A. nidulans* is a more cosmopolitan species and has been isolated from a variety of substrates including dead plant matter (Klich 2002). Both *N. crassa* and *A. nidulans* have a wide array of genomic, genetic, and biochemical tools that make these two species an ideal platform for comparative analyses of processes associated with plant cell wall deconstruction.

The production of hydrolytic plant cell wall degrading enzymes in filamentous fungi, including *N. crassa* and *A. nidulans* is dependent upon a release from carbon catabolite repression (CCR) as well as induction in response to the presence of plant cell wall material (Ruijter and Visser 1997; Mach and Zeilinger 2003; Aro et al. 2005; Andersen et al. 2008; Portnoy et al. 2011; Sun and Glass 2011; Zhou et al. 2012). In a previous study, we identified a zinc binuclear cluster transcription factor, CLR-2 (NCU08042), that is essential for induction of genes encoding cellulases (Coradetti et al. 2012); the expression of *clr-2* is induced in the presence of plant cell walls (*Miscanthus*), cellulose, or cellodextrins (Tian et al. 2009; Znameroski et al. 2012). An *N. crassa* strain containing a deletion of *clr-2* is unable to utilize cellulose as a sole carbon source and fails to induce ∼2/3 of the genes identified in the “Avicel regulon” (Coradetti et al. 2012). *clr-2* is highly conserved in filamentous ascomycete fungi; a strain carrying a deletion of the *A. nidulans* ortholog of *clr-2* (*clrB)* also failed to induce a number of cellulolytic genes and lacked cellulase activity (Coradetti et al. 2012).

Here, we generated the first set of RNA-seq data from both wild-type *A. nidulans* and a Δ*clrB* mutant in response to carbon starvation or crystalline cellulose. In-depth comparative analyses of these data with published *N. crassa* wild type and Δ*clr-2* data unraveled both conserved and divergent genes associated with the CLR-2 versus the ClrB regulons in these two distantly related species. Importantly, we found that misexpression of *clr-2* in *N. crassa* induced cellulase gene expression and activity under noninducing conditions and also increased cellulase activity when the misexpression strain was exposed to crystalline cellulose. To our knowledge, this is first report of that the manipulation of one gene can circumvent the dependence of inducers for cellulase production. Our experiments suggest that the manipulation of *clr-2* orthologs among filamentous fungi holds great potential for the identification and characterization of new enzymes. These studies will increase our understanding of mechanistic aspects of plant cell wall deconstruction and the development of optimal enzyme cocktails for cellulose degradation.

## Experimental Procedures

### Strains

The wild-type *N. crassa* reference strain and background for mutant strains were OR74A (FGSC 2489) (Colot et al. 2006). The deletion strains for *clr-2* (FGSC 15,835) were obtained from the Fungal Genetics Stock Center (http://www.fgsc.net/) (McCluskey 2003). The *clr-2* misexpression strain was constructed by transforming a *his-3 rid-1 Δsad-1;* Δ*clr-2 A* mutant with a variant of plasmid pMF272, where the open reading frame and 3′ untranslated region (UTR) of *clr-2* were placed under the control of the promoter and 5′ UTR of the clock-controlled gene 1 (*ccg-1*). Transformants were selected for histidine prototrophy and backcrossed to a *his-3;* Δ*clr-2 A* strain to obtain a *his-3::pccg-1-clr-2 rid-1 Δsad-1;* Δ*clr-2 A* homokaryotic strain.

The *A. nidulans* reference strain was FGSC 4A. The Δ*clrB* deletion strain was constructed previously (Coradetti et al. 2012). The *clrB* misexpression strains were constructed by transforming a Δ*clrB* mutant (Δ*clrB*::*pyrG pyroA4 pyrG89*) with a DNA fragment consisting of the *Aspergillus fumigatus pyroA* gene, the *gpdA* (Punt et al. 1990) or the *alcA* (Waring et al. 1989) promoter and the coding region, and 3′ UTR of either *clrB* or *clr-2* inserted into the *pyrG* locus. Transformants were selected for pyridoxine prototrophy (Nayak et al. 2006). The DNA fragments were generated by fusion PCR and transformation was carried out as described previously (Szewczyk et al. 2006).

### Culture conditions

*N. crassa* cultures were grown on Vogel's minimal medium (VMM) (Vogel 1956); carbon sources were 2% w/v unless otherwise noted. Strains were inoculated into 3 mL VMM slants and grown at 30°C in the dark for 48 h, then at 25°C in constant light for 4–10 days to stimulate conidia production. Conidia were inoculated into 100 mL of liquid media at 10^6^ conidia/mL and grown at 25°C in constant light and shaking (200 rpm).

*A. nidulans* cultures were grown on *A. nidulans* minimal medium (MM) (Vishniac and Santer 1957). Carbon sources were 2% w/v unless otherwise noted. Conidia were grown on sucrose MM slants, then inoculated into 100 mL liquid media at 3 × 10^6^ conidia/mL and grown at 37°C in constant light and shaking (200 rpm). These culture conditions were optimal for cellulose utilization by each species in pilot experiments and are in line with previous studies on each species. The higher temperature and inoculum used for *A. nidulans* allowed for comparable biomass accumulation to *N. crassa* cultured over the same period, which was chosen as the best practical basis comparison between the two species.

### Media shift experiments

Cultures were grown 16 h on sucrose (or 1% glucose for the *A. nidulans* RNA-seq experiments), centrifuged at 2000 g for 10 min, and washed in VMM or MM (Vogel 1956) without a carbon source. Mycelia were resuspended in 100 mL VMM or MM with 2% carbon source (sucrose, cellulose [Avicel® PH-101, Sigma Aldrich, MO]) or with no carbon source added. Total RNA was extracted as described in Tian et al. (2009) at 4 h after shift for *N. crassa* cultures and 6 h after shift for *A. nidulans* cultures. These time points were selected as the earliest points of full cellulase gene induction by preliminary RT-PCR time course experiments. For enzyme activity assays, culture supernatants were sampled at 6, 12, 24, 36, 48, and 72 h (and 96 h for *A. nidulans*), centrifuged at 3000 g twice to remove mycelia, and stored at 4°C for prior to analysis. At the end of the time course experiments, fungal biomass was harvested, dried, and weighed. The relative proportion of mycelia to residual Avicel was calculated by solubilizing fungal biomass in boiling acetic nitric reagent as in Updegraff (1969), washing the residual mass thoroughly, then drying, and reweighing.

One mutant strain (Pg2) required alternate media switch conditions due to low growth and conidiation. Pg2 conidia were inoculated in 3 mL 1% glucose MM and grown 3 days at 37°C in constant light and shaking (200 rpm). Cultures were then washed in MM with no carbon and switched to 3 mL MM with 2% Avicel, 2% glucose, or no carbon source. Other *A. nidulans* strains used in RT-PCR experiments were cultured similarly, but with only 24 h pregrowth in 1% glucose to achieve similar biomass to Pg2.

### RNA sequencing and transcript abundance

Libraries were prepared with standard protocols from Illumina Inc (San Diego, CA) and sequenced on the Illumina Genome Analyzer IIx and HiSeq 2000 platforms. Sequenced libraries were mapped against predicted transcripts from the *N. crassa* OR74A genome (Galagan et al. 2003) (http://www.broadinstitute.org/annotation/genome/neurospora/MultiHome.html) or the *A. nidulans* FGSC A4 genome http://www.broadinstitute.org/annotation/genome/aspergillus_group/MultiHome.html (Galagan et al. 2005) with Tophat v2.0.4 (Langmead et al. 2009). Transcript abundance (Fragments per Kilobase exon length per Megabase mapped, FPKM) was estimated with Cufflinks v2.0.2 (Roberts et al. 2011) mapping against reference isoforms. Profiling data are available as a supplemental Data S1 and at the GEO (http://www.ncbi.nlm.nih.gov/geo/; Series Record GSE44100). Raw counts for reads mapping to unique exons were tallied with HTSeq (Anders and Huber 2010) and used as inputs for the DESeq software packages. Genes with a multiple hypothesis–adjusted p-value below 0.05 and at least fourfold induction on Avicel were selected as belonging to the Avicel regulon and used for further analyses.

Orthologous gene pairs were taken from the fungal orthogroups repository (http://www.broadinstitute.org/regev/orthogroups/) and supplemented by a best reciprocal blast search for recently added or revised gene models in the *N. crassa* and *A. nidulans* genomes. In few cases, Avicel-induced genes had an ortholog listed in the repository with very low expression and a second close homolog with similar sequence conservation which showed specific induction. In those cases, the induced homolog was selected as the likely ortholog. For each species, specifically induced genes without a clear ortholog were used as BLAST queries against the other genome. The most significant alignment (*E*-value <10^−50^) was taken as a possible paralog. In some cases this procedure matched several paralogs in one species to a single gene in the other. These pairings were treated as distinct entries for clustering analysis. Genes with very low expression (FPKM <5 on Avicel) were filtered from the cross-species clustering analysis. For clustering of orthologous and paralogous pairs of genes, FPKM were log transformed and normalized on a per gene basis such that the highest value recorded in any sample for either member of the pairing was set to 1. The normalized values were then scaled and centered to set 1 FPKM to −1 and the maximal FPKM to 1 for clustering. Average linkage clustering was performed with Cluster 3.0 (de Hoon et al. 2004) using Pearson correlation as the similarity metric.

For clustering of WT and mutant *clr-2/clrB* libraries in each species, FPKM were log transformed then normalized and centered on per gene basis with Cluster 3.0 standard settings, such that values from each gene ranged from −1 (minimum/uninduced value) to 1 (maximum/induced value) and clustered with *K*-means clustering with Euclidean distance as the similarity metric.

### Enzyme activity assays

CMCase and xylanase activity of culture supernatants was assayed with Remazolbrilliant Blue R-conjugated carboxymethyl cellulose (CMC) and xylan (birchwood) kits from Megazyme (Wicklow, Ireland). Briefly, in these assays enzyme activity is measured by the release of ethanol soluble short-chain dye-conjugated oligomers from the long-chain substrates, followed by quantification with a UV spectrophotometer. Total protein was determined with the Bradford assay from BioRad (Hercules, CA). Cellulase activity was measured as previously reported (Tian et al. 2009) with minor adjustments. A 200 μL aliquot of culture supernatant was added to a final volume of 1 mL 1% Avicel in 50 mmol/L sodium acetate and incubated with shaking at 37°C overnight. Released glucose and cellobiose levels were measured by high performance liquid chromatography.

### Variation and statistical significance

All error bars on charts and figures represent standard deviation from biological triplicate samples unless otherwise noted.

## Results

### A comparison of the Avicel regulons of *A. nidulans* and *N. crassa* reveals conserved clusters of genes

**Figure 1:**
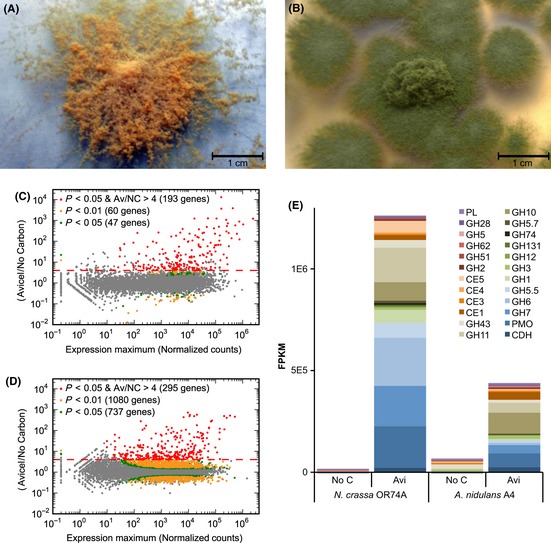
The Avicel regulons of *N. crassa* and *A. nidulans*. (A) Photograph of *N. crassa* grown on a Petri dish showing mycelia and conidia (orange). (B) Photograph of *A. nidulans* grown on a Petri dish showing mycelia and conidia (green). (C) Relative mRNA abundance of *N. crassa* exposed to Avicel versus no carbon conditions. Genes showing significant differential expression are colored by adjusted P-value. (D) Relative mRNA abundance of *A. nidulans* exposed to Avicel versus no carbon source conditions. Genes showing significant differential expression are colored by adjusted *P*-value. (E) Total expression from RNA-seq data of genes encoding the major classes of plant cell wall carbohydrate-active enzymes (CAZy; (Cantarel et al. 2009)) from a WT *N. crassa* culture shifted to media with no carbon source or to Avicel and from a WT *A. nidulans* culture shifted to media with no carbon source or to Avicel. FPKM (Fragments Per Kilobase per exon per Megabase mapped) for individual genes were averaged between biological replicates and pooled by CAZy class. Classes acting primarily on crystalline cellulose are shown in blue, classes acting primarily on hemicellulose are shown in brown and orange, and classes acting primarily on pectin are colored in purple.

To comprehensively compare the CLR-2/ClrB regulons in these two evolutionarily divergent species, we first characterized the transcriptional response of *A. nidulans* to Avicel. To compare its Avicel regulon with that of *N. crassa A. nidulans* wild-type strains were switched from a simple carbon source, glucose to Avicel, or no carbon source media; transcriptional profiles of the switched cultures were analyzed with RNA-seq. Genes strongly induced on Avicel (>fourfold transcript abundance with a statistical significance beyond a 5% false discovery rate, as calculated with DESeq) were compared between the two species (Fig. 1; Data S1).

**Figure 2:**
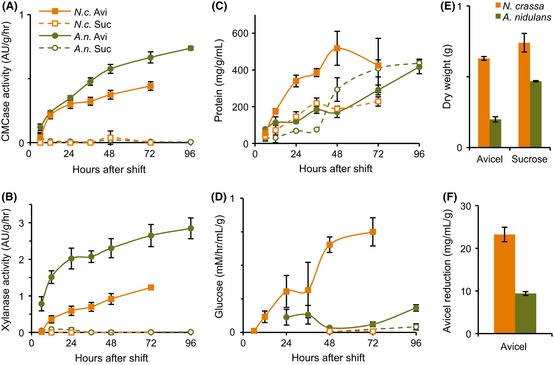
Time course of enzyme activity from *N. crassa* and *A. nidulans* culture supernatants. (A) CMCase activity of culture supernatants from *N. crassa* or *A. nidulans* cultures shifted to either Avicel or sucrose, as measured by cleavage of dye-conjugated CMC. (B) Xylanase activity of culture supernatants from *N. crassa* or *A. nidulans* cultures shifted to either Avicel or sucrose, as measured by cleavage of dye-conjugated xylan. (C) Secreted protein in *N. crassa* or *A. nidulans* supernatants from cultures shifted to either Avicel or sucrose, as determined by Bradford assay. (D) Cellulase activity, as determined by sugar release from Avicel, of *N. crassa* or *A. nidulans* supernatants from cultures shifted to either Avicel or sucrose. (E) Final mycelia dry weights 72 and 96 h after shift of *N. crassa* and *A. nidulans* cultures to sucrose or Avicel. Biomass in Avicel cultures was estimated by degrading mycelia and weighing the recalcitrant fraction (Updegraff 1969). (F) Cumulative breakdown of Avicel normalized to final dry weight of cultures. (A–D) All data points normalized to final culture biomass as in (E).

Both *N. crassa* and *A. nidulans* exhibited a broad and similar transcriptional response to Avicel with several hundred genes meeting the conservative criteria for specific induction (Fig. 1C, D). The magnitude of this induction was greater in *N. crassa*, however, particularly for enzyme classes with predicted function in the degradation of crystalline cellulose, such as genes encoding enzymes in the glycoside hydrolase (GH; (Cantarel et al. 2009)) 7 family (cellobiohydrolase I), GH6 family (cellobiohydrolase II), polysaccharide monooxygenases (formerly GH61 family), and GH5 family (endoglucanases) (Fig. 1E). To compare enzyme activity and protein secretion, supernatants from *N. crassa* and *A. nidulans* cultures shifted to either Avicel or sucrose were evaluated. Enzyme activity on amorphous cellulose (carboxymethyl cellulose) and xylan was significantly higher from *A. nidulans* Avicel-grown supernatants as compared with *N. crassa* (Fig. 2A, B). However, enzyme activity on crystalline cellulose was significantly higher for Avicel supernatants from *N. crassa* (Fig. 2D, F). *N. crassa* also secreted more protein, consumed more cellulose, and accumulated more biomass on Avicel than *A. nidulans* (Fig. 2C, E). These results reflected the observed transcriptional differences between *N. crassa* and *A. nidulans* in genes encoding enzymes with predicted activity on crystalline cellulose (Fig. 1E).

**Figure 3:**
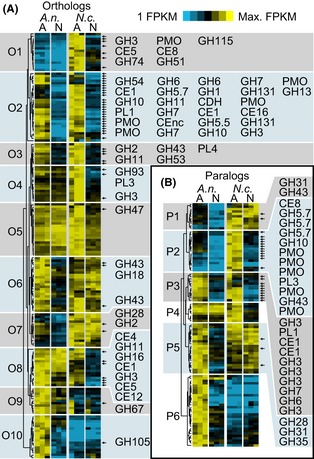
Hierarchical clustering of orthologous and paralogous genes induced by exposure to Avicel in *N. crassa* and *A. nidulans*. (A) Hierarchical clusters of FPKM for ortholog pairs (O1-O10) for which at least one member passed criteria for specific induction under either Avicel [A] or no carbon source [N] conditions. *A.n. *= *Aspergillus nidulans; N.c. = Neurospora crassa*. (B) Hierarchical clusters of FPKM for paralogous pairs (P1-P6) of genes induced under Avicel [A] or no carbon source [N] conditions (see methods for details on paralog selection). Heat maps display log (FPKM) scaled from 1 (bright blue) to the maximal value for each pair (bright yellow). CAZy genes in each cluster are marked with small arrows and listed in order to the right of each cluster.

To assess the conservation of the Avicel regulon between *N. crassa* and *A. nidulans* on a gene-by-gene basis, expression of orthologous and paralogous gene pairs was sorted with hierarchical clustering. Among genes with likely orthologs in each species, 10 gene expression clusters were identified (Fig. 3; Data S1). Cluster O1 consisted of genes strongly induced in *N. crassa* under Avicel conditions, but with very low expression in *A. nidulans*. This cluster included genes encoding two putative transporters (NCU01633/AN4277 and *cdr4* NCU05591/AN0771), a β-glucosidase *gh3-6/bglB* (NCU07487/AN0712), as well genes encoding six additional secreted carbohydrate-active enzymes (CAZy; http://www.cazy.org), an acetylcholine esterase (NCU08752/AN7407), and a monooxygenase (NCU7055/AN8199).

Cluster O2 consisted of 29 genes strongly and specifically induced on Avicel in both species, 25 of which encoded secreted CAZy proteins (Data S1). These included the most highly expressed and well-characterized cellulases: *cbh-1* (NCU07340, orthologous to both *cbhA* AN5176 and *cbhB* AN0494), *gh6-2* (NCU09680 orthologous to both *cbhC* AN5282 and *cbhD* AN1273), *gh5-1/eglA* (NCU00762/AN1285), and multiple polysaccharide monooxygenases (PMO). Cluster O3 consisted of 9 genes strongly induced on Avicel, but with higher expression under starvation conditions than genes in cluster O2 (Data S1). Most of these genes are implicated in pectin or hemicellulose deconstruction. Also included were two transporters: cellodextrin transporter *cdt-1* (NCU00801/AN2814) and a less strongly expressed putative sugar transporter NCU06384/AN3498.

Clusters O4 and O5 consisted of genes with specific induction under Avicel conditions in *N. crassa*, but relatively constant expression at an intermediate level in *A. nidulans*. These clusters were differentiated mostly by their relative transcript abundances in *N. crassa* under starvation conditions. They included several genes that function in xylose utilization: xylose reductase NCU08384/AN0423, xylitol dehydrogenase NCU00891/AN9064, xylulose kinase NCU11353/AN8790, ribose-5-phosphate isomerase NCU10107/AN5907, mitochondrial triose phosphate isomerase NCU10106/AN5908, and a xylose-specific transporter NCU06138/AN0250 (Du et al. 2010). Six genes with known or suspected roles in protein secretion and trafficking also clustered tightly in O5: *hsp70-6* NCU09485/AN0847, calreticulin/calnexin homolog *clxA* NCU09265/AN3592, *sec61-1* NCU08897/AN7721, *grp78/bipA* NCU03982/AN2062, *SEC62* homolog NCU06333/AN6269, and protein disulfide isomerase A *pdiA* NCU09223/AN7436. Also prominent in these clusters were several genes involved in aspartate/asparagine and aromatic amino acid biosynthesis. The cellulolytic regulators *clr-1/clrA* (NCU07705/AN5808) and *clr-2/clrB* also clustered in O5 and O4, respectively. They showed specific induction in both species, but the magnitude of the change was more dramatic in *N. crassa*.

Clusters O6 and O7 (Data S1) consisted of 23 and 14 genes, respectively, with specific induction under Avicel conditions in *A. nidulans*, but with relatively constitutive expression in *N. crassa*. They included a few secreted pectinases (arabinosidase *gh43-7* NCU05965/AN7781, exopolygalacturonase *gh28-2* NCU06961/AN8891, GDSL lipase/acylhydrolase NCU06364/AN1792) and intracellular enzymes with activity on pectin breakdown products (L-galactonate dehydratase NCU07064/AN6035, β-xylosidase *gh43-5/bxlD* NCU09652/AN7864, β-galactosidase *gh2-3/lacD* NCU00810/AN3201, and UDP-glucose 4-epimerase NCU05133/AN2951).

Cluster O8 had generally higher expression in *A. nidulans*, but more specific induction under Avicel conditions in *N. crassa*. It was most dominated by genes encoding predicted pectinases and hemicellulases (rhamnogalacturonan acetylesterase *ce12-1* NCU09976/AN2528, *faeC* NCU08785/AN5267, acetylxylan esterase *ce5-3* NCU09664/AN5309, *gh11-1/xlnA* NCU02855/AN3613, and mixed-linked glucanase NCU01353/AN0245) and hypothetical proteins. It also included the major secreted β-glucosidase *gh3-4/bglL* NCU04952/AN2828. Clusters O9 and O10 were induced in *A. nidulans*, but not in *N. crassa*. Cluster O9 included several stress response genes (hsp30-like proteins AN3555 and AN2530 orthologous to *hsp30* NCU09364, *hsp78* NCU02630/AN1163, a MFS multidrug transporter NCU00306/AN0732, and a mitochondrial hypoxia-responsive gene NCU02623/AN1066).

Genes with uncertain orthology relations were matched as pairs of cross-species paralogs and clustered by FPKM with the same parameters as the orthologous pairs, yielding 6 major clusters (Fig. 3B; Data S1). Most of the genes encoding CAZy proteins in this set clustered together in P2 and P3. Genes in P2 showed some induction in both species, but with greater magnitude in *N. crassa*. Most notable in this group were an expansion of highly expressed PMOs in *N. crassa* and an expansion of endo-β-mannanases (GH5.7) in *A. nidulans*. Genes in cluster P3 were comparably expressed in both species. Most prominent in P3 were several β-glucosidases and β-xylosidases and the highly expressed CBHII homolog *gh6-3* NCU07190, most closely related to *cbhD* AN1273. Cluster P5 genes were induced on Avicel in *A. nidulans*, and included 3 paralogous genes encoding enzymes associated with hemicellulose and/or pectin breakdown. Cluster P6 included *A. nidulans* paralogs that were induced under Avicel conditions, but not in *N. crassa*.

Polysaccharide monooxygenases (GH61) oxidatively cleave cellulose (Phillips et al. [Bibr b49][Bibr b50]; Quinlan et al. 2011; Beeson et al. 2012). Based on phylogenetic analyses, three PMO enzyme classes have been identified in *N. crassa* (Li et al. 2012); these genes had a particularly distinct pattern of expression between the two species. In *N. crassa*, 10 of 14 predicted PMO genes met our criterion for specific induction on Avicel, with no particular gene accounting for more than 27% of total FPKM among this class. In *A. nidulans,* 5 of 9 predicted PMO genes were specifically induced, but just one type-3 PMO, AN3860, accounted for more than 93% of total FPKM under Avicel conditions.

*N. crassa* genes without clear homologs in *A. nidulans* that were induced on Avicel were dominated by hypothetical proteins (34 of 39; Data S1); 12 were predicted to be secreted and may have novel functions in plant cell well deconstruction. *A. nidulans* genes without clear homologs in *N. crassa* that were induced on Avicel included genes encoding predicted pectinases and enzymes with roles in secondary metabolism (sterigmatocystin/aflatoxin, terrequinone A, and penicillin biosynthesis).

### Conservation of gene regulation by CLR-2 and ClrB

**Figure 4:**
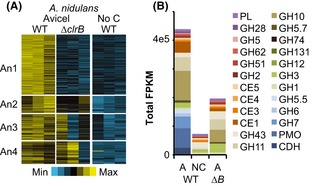
Dependence of Avicel-induced genes on functional ClrB in *A. nidulans*. (A) K-means clusters of Avicel-induced genes in an *A. nidulans* Δ*clrB* mutant strain on Avicel as compared to a WT strain on Avicel or media with no carbon source. Heat map displays log (FPKM) scaled from minimal expression (bright blue) to the maximal expression (bright yellow). (B) Total expression of genes encoding major CAZy classes in the Δ*clrB* mutant on Avicel as compared to a WT strain on Avicel or media with no carbon source. Note the absence of expression of genes encoding predicted cellulases in the Δ*clrB* mutant (PMO, GH6, GH7, and GH5.5 classes, shown in blue).

To assess conservation of function and respective target genes of CLR-2 and ClrB*,* we analyzed expression patterns of the Avicel regulon (295 genes total) in WT *A. nidulans* transferred to Avicel or no carbon source media versus the Δ*clrB* mutant transferred to Avicel. We identified 4 clusters of genes in *A. nidulans* (An1-An4; Fig. 4A; Data S1). Cluster An1 was comprised of 141 genes strictly dependent on *clrB*. It was dominated by highly expressed genes encoding the core enzymes associated with crystalline cellulose degradation: the CDH, PMO, GH7, GH6, and GH5.5 families. Although genes encoding cellulases were not induced in Δ*clrB* on Avicel, expression of some genes associated with hemicellulose degradation showed an increased expression level in the *ΔclrB* mutant on Avicel over WT under no carbon conditions (Fig. 4B).

Cluster An2 was composed of 40 genes with intermediate transcript abundance in Δ*clrB* strains on Avicel as compared with WT, indicating a partial or indirect requirement for functional ClrB for induction on Avicel (Data S1). This cluster included several genes with predicted function in secondary metabolite biosynthesis, including several robustly expressed genes of the sterigmatocystin biosynthesis gene cluster. Genes implicated in monodictyphenone and penicillin biosynthesis and a putative polyketide synthase (AN12331) also showed *clrB*-dependent induction, but were expressed at lower levels.

Genes in clusters An3 and An4 (61 and 53 genes) reached full induction in Avicel in the Δ*clrB* mutant, although variance in expression was high between biological replicates (Fig. 4; Data S1). This cluster was dominated by expression of the GH10, GH11, GH43, and GH62 gene families, which encode the major enzyme classes dedicated to hemicellulose degradation. Several of these genes or their orthologs have been identified as targets of the transcriptional regulator XlnR in *Aspergilli* (van Peij et al. 1998), and also had variable expression in *A. nidulans* when induced by xylose (Andersen et al. 2008).

The genes identified as ClrB dependent in *A. nidulans* were compared with those identified as CLR-2 dependent in *N. crassa* in response to Avicel (Coradetti et al. 2012). Twenty-eight orthologous pairs of genes with conserved regulation by CLR-2/ClrB were identified (Table 1). All of these genes encode proteins with some functional annotation (no hypothetical), although some are based solely on homology to enzymes with limited experimental data. The majority encoded secreted proteins that represent the conserved core of enzymes for cellulose degradation, including well-studied endoglucanases, exoglucanases, and β-glucosidases, as well as the newly characterized polysaccharide monooxygenases. The conserved list also included xylan and pectin degrading enzymes, including NCU00449 and NCU09764, orthologs to AN7598 (GH131; β-glucanase), and which have sequence similarity to a characterized glucuronan lyase from *T. reesei* (Konno et al. 2009). A conserved CLR-2/ClrB target NCU07143/AN2112 is homologous to a recently characterized extracellular aldonolactonase from *M. thermophila* (Beeson et al. 2011) and was identified in *N. crassa* secretome studies on Avicel (Phillips et al. 2011ab2011b). The expression of genes encoding several intracellular enzymes had conserved modulation by CLR-2/ClrB, including an intracellular β-glucosidase (NCU00130) and a β-mannosidase (NCU00890), as well as the cellodextrin transporter, *cdt-1* (NCU00801) (Galazka et al. 2010). Finally, one transcriptional regulator was conserved (*clr-1/clrA*). In *N. crassa clr-1* is required for the induction of *clr-2* in response to cellulose (Coradetti et al. 2012).

**Table 1:** Conserved orthologous genes in the CLR-2 and ClrB regulons

NCU#	Gene and annotation	CAZy	AN#	*Gene*
Orthologs with conserved dependence on *clr-2/clrB*				
NCU07340	*cbh-1* cellobiohydrolase 1	CBM1, GH7	AN5176	*cbhA*
			AN0494	*cbhB*
NCU09680	*gh6-2* cellobiohydrolase 2	CBM1, GH6	AN1273	*cbhD*
			AN5282	*cbhC*
NCU00762	*gh5-1* endoglucanase 2	CBM1, GH5.5	AN1285	*eglA*
NCU07760	*gh61-2* PMO	CBM1, PMO	AN3860	
NCU00206	*cdh-1* cellobiose dehydrogenase	CBM1, CDH	AN7230	
NCU07143	*lac-2* aldonolactonase		AN2112	
NCU08412	endo-β-1,4-mannanase	GH5.7	AN6427	*manC*
NCU03328	*gh61-6* PMO	GH61	AN6428	
NCU02344	*gh61-12* PMO	GH61	
NCU04952	*gh3-4* secreted β-glucosidase	GH3	AN2828	*bglL*
NCU09664	*ce5-3* acetylxylan esterase	CE5	AN5309	
NCU00449	glycoside hydrolase	GH131	AN7598	
NCU09764	*gh61-14* glycoside hydrolase	CBM1, GH131	
NCU06326	pectate lyase 1	PL1	AN0741	*pelA*
NCU03181	acetylxylan esterase	CEnc	AN5320	
NCU00972	arabinogalactan endo-1,4-β-galactosidase	GH53	AN5727	*gala*
NCU04997	*gh10-3* xylanase	CBM1, GH10	AN7401	*xlnE*
NCU07705	*clr-1* cellulose degradation regulator 1		AN5808	*clrA*
NCU05598	*asd-1* rhamnogalacturonase B	PL4	AN7135	*rglA*
NCU09976	*ce12-1* rhamnogalacturonan acetylesterase	CE12	AN2528	
NCU08176	*ply-2* pectate lyase A	PL3	AN6106	*plyF*
Orthologs with conserved modulation by *clr-2/clrB*				
NCU00130	*gh1-1* cytoplasmic b-glucosidase	GH1	AN10124	
NCU00801	*cdt-1* cellodextrin transporter		AN2814	
NCU04854	*gh7-2* endoglucanase	GH7	AN3418	*eglB*
NCU00890	*gh2-1* β-mannosidase	GH2	AN3368	*mndB*
NCU09416	cellulose-binding GDSL lipase/acylhydrolase	CBM1, CE16	AN6422	

### Inducer-independent expression of cellulases in *N. crassa*

Transcripts of *clr-2* and *clrB* accumulate under cellulolytic conditions (Coradetti et al. 2012). To test whether *clr-2* expression alone was sufficient for cellulase enzyme secretion in *N. crassa*, we placed a copy of the *clr-2* coding region under control of the promoter from clock-controlled gene 1 (P*ccg-1*) in a Δ*clr-2* (strain Pc2; see Experimental Procedures). Under our culture conditions of vegetative growth in constant light and temperature, circadian rhythm is dampened (Elvin et al. 2005) and P*ccg-1* is essentially a constitutive promoter.

**Figure 5:**
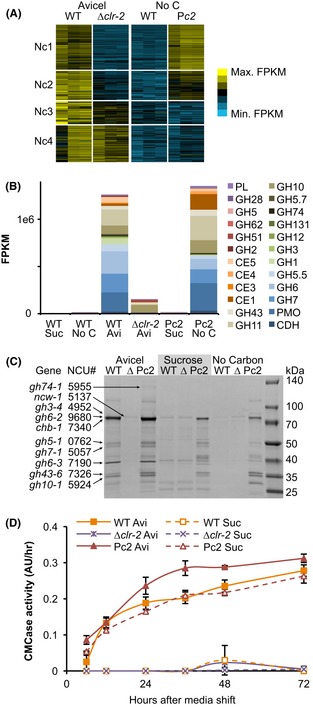
Cellulase gene expression and activity by misexpression of *clr-2* in *N. crassa*. (A) *K*-means clusters of genes (Nc1-4) induced under Avicel or no carbon conditions in WT, under Avicel conditions in a *Δclr-2* mutant and under no carbon media conditions for a strain with constitutive expression of *clr-2* (Pc2). Heat map displays log (FPKM) scaled from min expression (bright blue) to the maximal expression (bright yellow). (B) Total expression of genes encoding major CAZy classes of Pc2 on sucrose versus no carbon media, as compared to WT on sucrose, no carbon media, or Avicel. Note the similarity in CAZy expression class in WT on Avicel versus the Pc2 strain on media lacking a carbon source. A Δ*clr-2* strain on Avicel shows a low induction of genes encoding some hemicellulases, due to slight contamination of Avicel with hemicellulose (Znameroski et al. 2012). (C) Coomasie-stained SDS PAGE gel of culture supernatants from WT,*Δclr-2* (Δ), and Pc2 strains shifted to Avicel (4 days), sucrose (2 days), or media lacking a carbon source (2 days). Secreted proteins labeled from molecular weights and intensity patterns are derived from (Tian et al. 2009; Phillips et al. 2011ab2011b; Znameroski et al. 2012). Note similarity of secreted protein profile of the Pc2 strain grown on sucrose or no carbon as compared to Avicel-exposed WT and Pc2 cultures. (D) A time course of CMCase activity of supernatants from WT,*Δclr-2,* and Pc2 cultures after shift from sucrose pregrown cultures (16 h) to either Avicel or sucrose.

Expression patterns of the Avicel regulon (193 genes total) were compared in WT *N. crassa* on Avicel and no carbon media, the Δ*clr-2* strain on Avicel, and the Pc2 strain on no carbon media. Four major expression clusters were identified in WT (Fig. 5A; Nc1-Nc4); the Nc1 and Nc2 clusters were dependent upon functional *clr-2*. The Nc1 cluster (65 genes) was comparably or more strongly expressed in the Pc2 strain under noninducing conditions. This cluster was highly enriched for genes encoding secreted CAZy proteins and included most major cellulase genes (Data S1). These data indicate that it is possible to induce cellulase gene expression in the absence of an inducer in *N. crassa*. Under no carbon source conditions, transcript abundances of CAZy genes in the Pc2 strain were comparable with those observed in the WT strain shifted to Avicel (Fig. 5B). However, when Pc2 was transferred to fresh sucrose media, CCR strongly reduced CAZy expression, although it was elevated over no carbon conditions and far above that measured in the WT on sucrose.

The Nc2 cluster (42 genes) had expression an order of magnitude lower than WT in the Δ*clr-2* mutant on Avicel, but still had significant induction over the WT on no carbon. They also showed some induction in Pc2 on no carbon, but most did not reach the same abundances as in the WT on Avicel. These data suggest that the genes in Nc2 require another cellulose-dependent factor for full induction. The Nc2 cluster included one highly expressed cellobiohydrolase *gh6-3* (NCU07190), the major intracellular β-glucosidase *gh3-1* (NCU00130), and the cellodextrin transporter *cdt-1* (NCU00801).

The Nc3 cluster (33 genes) was fully to partially dependent on *clr-2* for induction under Avicel conditions, but remained uninduced by misexpression of *clr-2* in Pc2. This cluster was dominated by hypothetical proteins that had low expression levels, but notably included one β-glucosidase gene *gh3-4* (NCU07487). The Nc4 cluster (53 genes) was *clr-2* independent and was not induced by misexpression of *clr-2* in Pc2. This cluster was dominated by xylanases and the xylose utilization pathway genes, reflecting induction of these genes in a *clr-2*-independent manner by low concentrations of hemicellulose retained in Avicel (Znameroski et al. 2012).

When supernatants taken from Pc2 cultures 24 h after a shift to sucrose were analyzed with SDS PAGE, a similar protein pattern was observed to that seen in WT and Pc2 cultures shifted to Avicel (Fig. 5C). The secreted proteins were fully active, as demonstrated by CMCase activity of the sucrose shifted Pc2 supernatants (Fig. 5D). The misexpression of *clr-2* complemented the cellulose growth defect and cellulase gene induction in the *Δclr-2* strain under Avicel conditions and in fact, showed elevated levels of CMCase activity.

### Misexpression of *clrB* and *clr-2* in *A. nidulans*

**Figure 6:**
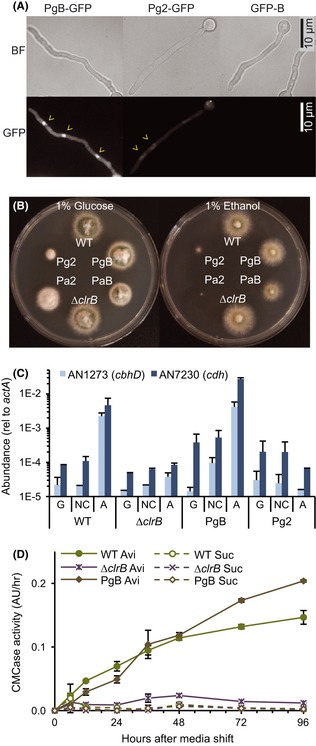
Phenotypes associated with misexpression of *clrB* or *clr-2* in *A. nidulans*. (A) Localization of GFP-tagged constructs in cells germinated in liquid glucose medium. GFP-labeled ClrB under native promoter (strain GFP-B) had no detectable fluorescence. GFP-labeled ClrB or CLR-2 driven by the *gpdA* promoter (strain GFP-PgB and GFP-Pg2) accumulated in nuclei, which were clearly distinguishable by their shape and prominent nucleoli. Arrowheads indicate nuclei. (B) WT and misexpression strains grown 3 days on 1% glucose or 1% ethanol plates at 30°C. PgB and Pg2: constitutive expression of *clrB* and *clr-2*, respectively, by the *gpdA* promoter in *ΔclrB* background. PaB and Pa2: ethanol-inducible expression of *clrB* and *clr-2,* respectively, by the *alcA* promoter in *ΔclrB* background. (C) Transcript abundance of two major cellulases (*cbhD* and *cdh*) in *A. nidulans*WT versus strains with constitutive expression of *clrB* (PgB) or *clr-2* (Pg2) when shifted to glucose [G], no carbon media [NC], or Avicel [A] for 6 h. Transcript abundance relative to *actA* was measured with real-time quantitative RT-PCR. (D) A time course of CMCase activity in WT, Δ*clrB,* and PgB strains following a shift to either Avicel or sucrose.

Our data indicated that it is possible to produce and secrete cellulases under noninducing conditions by heterologous regulation of the transcriptional regulator, CLR-2. To test whether misexpression of *clr-*2 homologs is sufficient for inducer-free cellulase production and secretion in other ascomycete fungi, we inserted the *clrB* coding sequences into the *pyrG* locus of the *A. nidulans* Δ*clrB* strain under the regulation of the constitutive *gpdA* promoter (PgB). A strain carrying an N-terminal GFP-tagged allele (GFP-PgB) showed nuclear GFP fluorescence on glucose liquid medium (Fig. 6A); *clrB* driven by its own promoter showed no fluorescence (Fig. 6A; GFP-B). The *gpdA*-regulated expression of *clrB* restored cellulase gene induction, growth, and enzyme activity to the Δ*clrB* mutant on Avicel (Fig. 6C, D). Similar to the *N. crassa clr-2* misexpressed strain, the *A. nidulans* PgB strain showed higher CMCase activity over the 96-hr time course as compared with WT. However, CMCase activity was not significantly induced when the PgB strain was grown for extended periods of time under sucrose conditions (Fig. 6D). Consistent with this result, mRNA abundance for two ClrB-dependent genes was several orders of magnitude below that of a WT culture shifted to Avicel when the PgB strain was shifted from sucrose to no carbon conditions (Fig. 6C), although AN7230 (CDH) transcript abundance was detectably higher in PgB strain than the WT under no carbon conditions. These data indicate that, in contrast to *N. crassa*, misexpression of *clrB* in *A. nidulans* was not sufficient to induce WT levels of cellulase gene expression and enzyme activity.

We then tested whether the introduction of *clr-2* could complement the Δ*clrB* mutant for the induction of cellulolytic genes under Avicel conditions. As for *clrB*, the *clr-2* coding sequence was placed under the control of the *gpdA* (Pg2) or *alcA* (Pa2) promoter. An N-terminal GFP-tagged Pg2 allele showed nuclear localization during growth on glucose in the Δ*clrB* mutant (Fig. 6A). However, *clr-2* was unable to complement the Δ*clrB* mutant for the induction of cellulolytic genes under Avicel conditions. Induction of cellulase gene expression under noninducing conditions was also not observed (Fig. 6C). In fact, the Pg2 (*gpdA-clr-2*) showed a strong growth defect under all conditions (Fig. 6B), whereas the strain with *clr-2* under control of the ethanol-inducible *alcA* promoter (Pa2) was severely deficient for growth on inducing ethanol media. In contrast, an ethanol-inducible *clrB* strain (PaB) grew well in both conditions, although detectably slower than WT on ethanol.

## Discussion

In all filamentous fungi studied to date, expression of cellulolytic genes requires both relief from CCR and the presence of an inducer molecule, such as cellobiose or cellodextrins, or in *T. reesei*, cellobiose, sophorose, lactose, or xylose (Sternberg and Mandels 1979; Stricker et al. 2006, 2007; Hakkinen et al. 2012), or xylose in the Aspergilli (Gielkens et al. 1999; Andersen et al. 2008; Noguchi et al. 2009). Several independent lines of mutagenesis have produced strains with increased cellulase production and decreased CCR (Peterson and Nevalainen 2012; Liu et al. 2013; Porciuncula et al. 2013), but constitutive, robust production of cellulases without inducing molecules has remained elusive over 30 years of mutagenesis-based research. Our data show that it is possible to produce and secrete cellulases under noninducing conditions by misexpression of the transcriptional regulator, CLR-2, the first report of a transcription factor necessary and sufficient for cellulase activity in any cellulolytic filamentous fungus. This observation suggests new opportunities for optimization of cellulase enzyme production in industrial strains without the need for cellulosic substrates or other inducing molecules. A genetic “master switch” for cellulase secretion may be particularly useful in the many species for which there are no known soluble cellulase-inducing molecules and for which limited genetic tools make extensive genetic engineering impractical.

Misexpression of *clr-2* in *N. crassa* resulted in inducer-independent production of plant cell wall degrading enzymes. In *A. nidulans,* misexpression of *clrB* produced no detectable enzyme activity under noninducing conditions, although RT-PCR analyses revealed a measurable increase in transcript abundance for a cellobiose dehydrogenase (AN7230). We therefore infer that there is a fundamental difference in cellulase gene induction between *Neurospora* and *Aspergillus* species. Whereas CLR-2 is fully competent to drive transcription of its targets in noninducing conditions in *N. crassa*, ClrB requires other factors for any significant effect. These observations beg the question: are differences in mechanisms of CLR-2/ClrB induction indicative of an ancient divergence between Sordariomycete and Eurotiomycete fungi? Or are they reflective of the different lifestyles and preferred substrates of these two species? The DNA binding and dimerization domain of the N-terminus of CLR-2 and ClrB show several regions of sequence divergence, which are conserved broadly in Sordariomycete versus Eurotiomycete species. The inability of *clr-2* to complement a deletion of *clrB* in *A. nidulans* and differences in CLR-2/ClrB amino acid sequence suggests divergence in regulatory mechanisms for cellulolytic genes.

Until recently, studies on regulation of plant cell wall deconstruction in *Aspergillus* species focused predominantly on induction by hemicellulosic sugars via the transcriptional regulator, XlnR (van Peij et al. 1998; Gielkens et al. 1999; Delmas et al. 2012). *A. nidulans* is more proficient at saccharification of xylan and relatively less effective at degrading crystalline cellulose than *N. crassa*. This phenotypic distinction was consistent with both relative expression and diversity of CAZy families between species, with *A. nidulans* specifically inducing more xylanases, β-mannases, and pectate lyases in particular. Together these data suggest that *A. nidulans* and perhaps other *Aspergillus* species have evolved to primarily utilize amorphous polymers over crystalline cellulose. Canonical cellulases may therefore serve a more accessory role for hemicellulolytic activity by synergistic action on complex substrates. In contrast, *N. crassa* has apparently evolved to rely more heavily on breakdown of crystalline cellulose via signaling to CLR-2. This regulatory aspect is likely conserved among members of the Sordariomycetes, as, similar to *N. crassa* (Sun et al. 2012), Δ*xlnR* mutants of *Fusarium graminearum* and *F. oxysporum* are unable to utilize hemicellulose, but are unaffected in cellulose deconstruction (Brunner et al. 2007; Calero-Nieto et al. 2007).

Clustering analysis of transcript abundance in an *N. crassa* strain with constitutive expression of *clr-2* suggests two modes of induction by CLR-2, one in which it acts independently and one in which it requires another cellulose-responsive factor to achieve full induction. In *T. reesei* and *Aspergilli,* the transcriptional regulator XYR1/XlnR, respectively, regulates expression of hemicellulase and cellulase genes (Stricker et al. 2006; Mach-Aigner et al. 2008; Noguchi et al. 2009). In *T. reesei*, a nonconserved transcriptional activator, ACEII (Aro et al. 2001) as well as the repressor ACEI (Aro et al. 2003) also affect cellulase gene expression. Recently, several additional transcription factors have been reported to play a role in regulation of plant cell wall degrading enzymes in ascomycete fungi, including BglR (*T. reesei*), which may influence CCR through regulation of genes encoding β-glucosidase enzymes (Nitta et al. 2012), ClbR (*A. aculeatus*), which is involved in XlnR-independent cellobiose and cellulose induction of *cbh1* (GH7) and endoglucanase (*cmc2*) (Kunitake et al. 2012), and McmA (*A. nidulans*) which affected induction of endoglucanases (eglA and eglB) and at least one cellobiohydrolase (cbhA) (Yamakawa et al. 2013). The interactions of these factors with each other and with XLR-1/XlnR and CLR-2/ClrB homologs deserve further investigation. An understanding of conservation of function across the phylogenetic spectrum may provide insight into fungal interaction with the environment and guide the selection and manipulation of fungal host species for industrial enzyme production.

Our comparative transcriptional analysis of *clr-2/clrB* mutants revealed a conserved and essential role for these transcription factors in response to cellulose. Strict conserved dependence on CLR-2/ClrB was limited to a relatively small group of orthologs, indicating a relatively simple cohort of essential genes for broad-spectrum cellulose degradation. The conservation of regulation of this gene set between two distantly related saprotrophic fungi suggests that these enzymes/proteins are key to cellulose deconstruction strategies. Recently, a transcriptional regulator (ManR) of beta-mannan utilization in *Aspergillus oryzae* was described (Ogawa et al. 2012). ManR is, in fact, the *A. oryzae* ortholog of ClrB and was recently shown to be essential for the regulation of cellulolytic genes (Ogawa et al. 2013). These results further confirm a conserved role for Clr-2/ClrB in regulation of synergistic ensemble of enzymes in response to plant cell wall polysaccharides in diverse ascomycete fungi.

A large group of genes encoding CAZy proteins within the CLR-2/ClrB regulons showed low sequence conservation or very low expression in either *N. crassa* or *A. nidulans*. These observations are consistent with the large variability in numbers and types of genes encoding predicted plant cell wall degrading enzymes in the genomes of filamentous ascomycete species (Espagne et al. 2008; Berka et al. 2011; Hakkinen et al. 2012). These genes likely serve accessory functions in plant cell wall degradation that may be specific to a particular species' ecological niche. Interestingly, we also observed coregulation of secondary metabolism genes with cellulolytic genes and which were partially dependent upon functional ClrB. Further comparisons of gene regulation across a wider panel of fungal species with varied ecological niches and different preferred substrates may provide insights into the adaptive advantages conferred by proteins/enzymes encoded by these genes.

The polysaccharide monooxygenase enzyme class is an example of divergent regulation across these species. Our data in *A. nidulans* and published data on *A. niger* indicate that a single gene of the type-3 PMO subclass dominates expression in *Aspergillus* species exposed to cellulose (this study) or wheat straw (Delmas et al. 2012). In contrast, a spectrum of type-1, type-2, and type-3 PMOs are expressed in *N. crassa* on Avicel and on bagasse for the closely related thermophilic industrial species, *M. thermophila* (Gusakov 2011; Visser et al. 2011) and *Thielavia terrestris* (Berka et al. 2011). This divergent regulation of PMOs may hold clues to an as-yet-unappreciated functional difference in subclasses of these enzymes; a recent study identified distinct product chemistries from a type-1 and type-3 PMOs (Bey et al. 2013). Comparative analyses of enzyme cocktails in diverse filamentous fungi enabled by transcriptomic and proteomic approaches, which in some cases will be expedited by misexpression of CLR-2 homologs, will advance efforts to define tailored enzyme mixtures for maximal efficiency on different lignocellulosic feedstocks for biofuels and production of value-added chemicals.

## Supporting Information

Data S1 Excel spreadsheet of expression profiling data from Neurospora crassa and Aspergillus nidulans.
